# Characterization of the complete chloroplast genome of toad lily *Tricyrtis Macropoda* (Liliaceae)

**DOI:** 10.1080/23802359.2018.1431069

**Published:** 2018-01-26

**Authors:** Rui-Hong Wang, Jing Gao, Xue Wu, Meng-Di Li, Chao Shen, Jun-Jie Wu, Wen-Di Yu, Jin-Liang Liu, Zhe-Chen Qi, Zong-Suo Liang

**Affiliations:** a College of Life Sciences, Zhejiang Sci-Tech University, Hangzhou, China;; b Zhejiang Province Key Laboratory of Plant Secondary Metabolism and Regulation, Hangzhou, China

**Keywords:** Chloroplast genome, Endangered, phylogenomics, *Tricyrtis*

## Abstract

*Tricyrtis* (Liliaceae) is an endemic genus in East Asia. Many of the species in the genus are in Endangered condition due to habitat loss and extensive horticultural usage in recent decades. In present study, we reported the first *Tricyrtis* chloroplast (cp) genome, *Tricyrtis macropoda*, based on Illumina pair-end sequencing data. The complete chloroplast genome size is 155,778 bp. In total, 131 genes were identified, including 85 protein-coding genes, 8 rRNA genes, and 38 tRNA genes. Fifteen genes are containing introns (*clpP* and *ycf3* contained two introns) and 14 genes had two copies. The overall GC content of this genome was 37.4%. A further phylogenomic analysis of Liliales, including 62 taxa, was conducted for the placement of genus *Tricyrtis*. The complete plastome of *T. marcropoda* will provide a valuable resource for further genetic conservation, phylogenomic, and evolution studies in the genus and family.

*Tricyrtis* Wall. is an endemic genus in East Asia, consisting of ca. 20 known taxa (Takahashi 1987; Hong and Jury 2012), with high ornamental value by showing attractive and sophisticated flowers (Mathew 1985). Due to extensive horticulture usage directly from wild resources and habitat loss by human activities in recent decades, most of the taxa of this group are listed as Threatened or Endangered. Here, we assembled and characterized the complete plastome of *Tricyrtis macropoda*. It is the first complete plastome reported in this genus, and will provide potential genetic resources for further evolutionary studies of the genus *Tricyrtis* and other relatives.

Total DNA was extracted from fresh leaves of *Tricyrtis macropoda* individual using DNA Plantzol Reagent (Invitrogen, Carlsbad, USA). It is collected from Mt. Qingshan, Zhejiang, China (Voucher No. WRH20170907, deposited at Zhejiang Sci-Tech University). The plastome sequences were generated using Illumina HiSeq 2500 platform (Illumina Inc., San Diego, CA, USA). In total, ca. 14.5 million high-quality clean reads (150 bp PE read length) were generated with adaptors trimmed. The CLC *de novo* assembler (CLC Bio, Aarhus, Denmark), BLAST, GeSeq (Tillich et al. 2017), and tRNAscan-SE v1.3.1 (Schattner et al. 2005) were used to align, assemble, and annotate the plastome.

The full length of *Tricyrtis macropoda* chloroplast genome (GenBank Accession No. MG599475) was 155,778 bp and comprised of a large single copy region (LSC with 85,175 bp), a small single copy region (SSC with 17,794 bp), and two inverted repeat regions (IR with 26,405 bp). The overall GC content of the *T. macropoda* cp genome was 37.4% and the GC content in the LSC, SSC, and IR regions are 35.3, 31.3, and 42.8%, respectively. A total of 131 genes were contained in the cp genome (85 protein-coding genes, 8 rRNA genes, and 38 tRNA genes. Fourteen genes had two copies, which included 3 PCG genes (*ndhB*, *rpl23*, and *ycf2*), 7 tRNA genes (*trnH-GUG*, *trnI-CAU*, *trnI-GAU*, *trnL-CAA*, *trnN-GUU*, *trnR-ACG*, and *trnV-GAC*), and all 4 rRNA species (*rrn4.5*, *rrn5*, *rrn16,* and *rrn23*). Among the protein-coding genes, two genes (*clpP* and *ycf3*) contained two introns, and other seven genes (*atpF*, *ndhA*, *ndhB*, *rpl2*, *rpoC1*, *rps12*, *rps16*) had one intron each.

Sixty-two chloroplast genome of Liliales were fully aligned with MAFFT v7.3 (Katoh and Standley 2013), and the maximum likelihood (ML) inference was performed using GTR + I + Γ model with 1000 bootstrap replicates with RAxML v.8.2.1 (Stamatakis 2014) on the CIPRES cluster service (Miller et al. 2010). The result revealed that well *Tricyrtis* placed at the basal position of Liliaceae with the current sampling extent (Figure 1).

**Figure 1.**
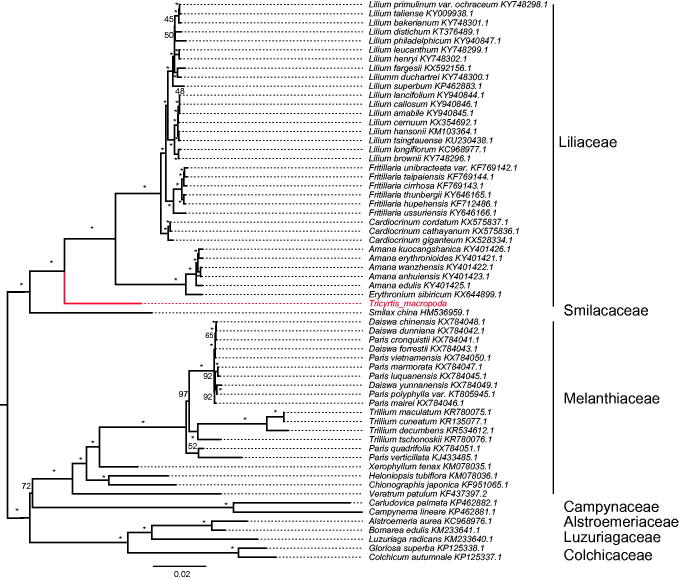
The best Maximum likelihood (ML) phylogram inferred from 62 chloroplast genomes in Liliales (bootstrap value are indicated on the branches, ‘*’ denotes a fully supported node).

## References

[CIT0001] Hong SWP, Jury SL. 2012 Phylogeny and molecular evolution of *Tricyrtis* (Liliaceae *s.l.*) inferred from plastid DNA *matK* spacer nucleotide sequences. J Plant Stud. 1:1–10.

[CIT0002] Katoh K, Standley DM. 2013 MAFFT multiple sequence alignment software version 7: improvements in performance and usability. Mol Biol Evol. 30:772–780. 23329690 10.1093/molbev/mst010 PMC3603318

[CIT0003] Mathew B. 1985 A review of the genus *Tricyrtis*. Plantsman. 6:193–224.

[CIT0004] Miller MA, Pfeiffer W, Schwartz T. 2010 Creating the CIPRES science gateway for inference of large phylogenetic trees. In: Proceedings of the gateway computing environments workshop (GCE). New Orleans, LA: p. 1-8.

[CIT0005] Schattner P, Brooks AN, Lowe TM. 2005 The tRNAscan-SE, snoscan, and snoGPS web servers for the detection of tRNAs and snoRNAs. Nucleic Acids Res. 33:W686–W689. 15980563 10.1093/nar/gki366 PMC1160127

[CIT0006] Stamatakis A. 2014 RAxML version 8: a tool for phylogenetic analysis and post-analysis of large phylogenies. Bioinformatics. 30:1312–1313. 24451623 10.1093/bioinformatics/btu033 PMC3998144

[CIT0007] Takahashi H. 1987 Distribution of *Tricyrtis* and its phytogeographical problems. Acta Phytotaxonomica Et Geobotanica. 42:113–124.

[CIT0008] Tillich M, Lehwark P, Pellizzer T, Ulbrichtjones ES, Fischer A, Bock R, Greiner S. 2017 GeSeq – versatile and accurate annotation of organelle genomes. Nucleic Acids Res. 45:W6–W11. 28486635 10.1093/nar/gkx391 PMC5570176

